# Frequent high-level expression of the immunotherapeutic target Ep-CAM in colon, stomach, prostate and lung cancers

**DOI:** 10.1038/sj.bjc.6602924

**Published:** 2006-01-10

**Authors:** P Went, M Vasei, L Bubendorf, L Terracciano, L Tornillo, U Riede, J Kononen, R Simon, G Sauter, P A Baeuerle

**Affiliations:** 1 Institute of Pathology, University Hospital Basel, Schönbeinstrasse 40, 4003 Basel, Switzerland; 2 Department of Pathology, Shiraz University of Medical Sciences, Shiraz, Iran; 3 Department of Pathology, University Hospital Freiburg, Freiburg i. Br., Germany; 4 Diomeda Life Sciences Inc., Rockville, MD, USA; 5 Institute for Pathology, University Clinic Hamburg-Eppendorf, Hamburg, Germany; 6 Micromet AG, Staffelseestr. 2, 81477 Munich, Germany

**Keywords:** Ep-CAM, colon cancer, stomach cancer, prostate cancer, lung cancer, monoclonal antibody

## Abstract

Epithelial cell adhesion molecule (Ep-CAM; CD326) is used as a target by many immunotherapeutic approaches, but little data are available about Ep-CAM expression in major human malignancies with respect to level, frequency, tumour stage, grade, histologic tumour type and impact on survival. We analysed by immunohistochemical staining tissue microarrays with 4046 primary human carcinoma samples from colon, stomach, prostate and lung cancers for both frequency and intensity of Ep-CAM expression under highly standardised conditions. A total of 3360 samples were analysable. High-level Ep-CAM expression was observed in 97.7% (*n*=1186) of colon, 90.7% of gastric (*n*=473), and 87.2% of prostate cancers (*n*=414), and in 63.9% of lung cancers (*n*=1287). No detectable Ep-CAM staining was found with only 0.4% of colon, 2.5% of gastric, 1.9% of prostate cancers, and 13.5% of lung cancers. The only significant correlation of Ep-CAM expression with tumour grading was observed in colon cancer where high-level Ep-CAM expression on grade 3 tumours was down to 92.1% (*P*<0.0001). Adenosquamous and squamous carcinomas of the lung had a lower percentage of high-level Ep-CAM expression compared to adenocarcinomas with 35.4 and 53.6%, respectively, and with 45.5 and 17.3% of tumours being Ep-CAM negative. With the exception of moderately differentiated colon carcinoma, where patients not expressing Ep-CAM on their tumours showed an inferior survival (*P*=0.0014), correlation of Ep-CAM expression with survival did not reach statistical significance for any of the other cancer indications and subgroups. In conclusion, the data strongly support the notion that Ep-CAM is a prime target for immunotherapies in major human malignancies. This is because the most common human cancers show (i) a low frequency of Ep-CAM-negative tumours, (ii) a high frequency of Ep-CAM expression on cells of a given tumour, and (iii) for most cancers, an insignificant influence of tumour staging, grading and histology on Ep-CAM expression.

Epithelial cell adhesion molecule (Ep-CAM)is a type I transmembrane glycoprotein of *M*_r_ 40 000 Da, expressed in most normal epithelial tissues on the basolateral surface. While no expression is seen on squamous epithelia and hepatocytes, it is detected on colon, gastric, prostatic and lung epithelium (Moldenhauer *et al*, 1987). Epithelial cell adhesion molecule is thought to function as a homotypic intercellular adhesion molecule (Litvinov *et al*, 1994). Its role in epithelial cell adhesion is dynamic and interconnected with E-cadherin (Litvinov *et al*, 1997). By upregulation of Ep-CAM, E-cadherin-mediated cell adhesion diminishes and the Ep-CAM mediated adhesion becomes predominant. During organogenesis in mice, Ep-CAM exhibits features of a morpho-regulatory molecule, which, for instance, is involved in the development of human pancreatic islets (Cirulli *et al*, 1998).

Epithelial cell adhesion molecule was discovered as one of the first tumour-associated antigens by immunising mice with human colon cancer cells followed by analysis of tumour-specific monoclonal antibodies (Herlyn *et al*, 1979; Sears *et al*, 1982). Epithelial cell adhesion molecule was then found to be expressed at a high level and frequency not only on colon cancer tissues but on most human adenocarcinomas (Went *et al*, 2004) as well as on squamous cell carcinomas (Quak *et al*, 1990). In the case of breast and ovarian cancers, Ep-CAM mRNA was found to be more than 100-fold overexpressed relative to normal epithelial tissues (Kim *et al*, 2003; Osta *et al*, 2004).

Overexpression of Ep-CAM is linked to differentiation and cell proliferation (Jordinson *et al*, 1999), although the molecular mechanism is still poorly understood. *In vitro*, its overexpression has been shown to be directly linked to stimulation of the cell cycle and proliferation by upregulating c-myc and cyclin A/E (Munz *et al*, 2004). In breast cancer cells, inhibition of Ep-CAM expression by small inhibitory RNA diminishes cell proliferation, migration and invasiveness of cells (Osta *et al*, 2004). Epithelial cell adhesion molecule gene expression appears to be negatively regulated by TNF-alpha through activation of NF-kappaB (Gires *et al*, 2001). Upon cell cycle arrest by various chemotherapeutics, Ep-CAM surface expression is enhanced (Flieger *et al*, 2001; Thurmond *et al*, 2003). As Ep-CAM is involved in adhesion, differentiation and cell proliferation, an influence of Ep-CAM expression on survival of cancer patients can be expected. In breast and gall bladder cancer, patients with high Ep-CAM-expressing primary tumours indeed showed a decreased survival (Gastl *et al*, 2000; Spizzo *et al*, 2002; Spizzo *et al*, 2004; Varga *et al*, 2004). In clear cell renal cell carcinoma, Ep-CAM expression is infrequent, but patients with Ep-CAM overexpressing tumours show a trend to better survival (Kim *et al*, 2004; Seligson *et al*, 2004; Went *et al*, 2005). A similar correlation was reported for gastric cancers (Songun *et al*, 2005). Very little information is currently available with regard to the correlation of Ep-CAM expression with survival and tumour staging for colon and lung cancers, while more recent studies have explored larger sample numbers of gastric (Songun *et al*, 2005) and prostate cancers (Poczatek *et al*, 1999; Zellweger *et al*, 2005) for Ep-CAM expression.

Due to its frequent and high-level expression, Ep-CAM was selected as target antigen for a multitude of immunotherapeutic approaches (Balzar *et al*, 1999). These include murine and human monoclonal antibodies, antibody conjugates with bacterial toxins and chemotherapeutics, and vaccines. Currently, a number of Ep-CAM-specific immunotherapies are in phase I and II clinical trials. These are anti-Ep-CAM antibodies ING-1 (de Bono *et al*, 2004), adecatumumab (Naundorf *et al*, 2002; Prang *et al*, 2005), and edrecolomab (Himmler *et al*, 2003), as well as an immunotoxin (Zimmermann *et al*, 1997; Di Paolo *et al*, 2003). It is therefore very important to understand which human cancers are amenable to Ep-CAM-specific immunotherapy based on Ep-CAM expression with respect to intensity, frequency and disease stage. Likewise, it is interesting to investigate a correlation of Ep-CAM expression with survival prognosis in patients.

The aim of the present retrospective study was to investigate the frequency and intensity of Ep-CAM expression in four major human cancers by the use of tissue microarrays. This technology allows for a simultaneous comparison of immunohistochemical staining patterns and intensities across a large panel of tumour samples. Variability due to fixation and staining procedures are reduced to a minimum, while comparability is maximised. Epithelial cell adhesion molecule expression results were correlated for the first time with clinico-pathological parameters in colon and lung cancers, while results from a large panel of gastric and prostate cancer samples are being compared to published data. Our results show that colon, gastric and prostate cancers as well as adenocarcinoma of the lung are promising indications for treatment with Ep-CAM-specific immunotherapies. Their frequencies of high-level Ep-CAM expression >80% may even obviate the need for prescreening of patients.

## MATERIALS AND METHODS

### Array composition

Primary tumours of colon, stomach, lung and prostate were included in this study. Formalin-fixed and paraffin-embedded tissue probes were retrieved from the archives of the Institute for Pathology of the University Hospital Basel (Switzerland), the City Hospital Triemli in Zürich (Switzerland) and the Departments of Pathology of the University of Freiburg (Germany) and the Shiraz University (Iran). Retrieval of tissue and clinical data was performed according to the regulations of the local institutional review board and data safety laws. The grade of the tumours was obtained by a review of each case by a specialised pathologist. A total of 1407 colon cancers (1261 adenocarcinomas NOS, five medullary carcinomas, 119 mucinous carcinomas, five signet ring carcinomas, four other types), 559 stomach, 1527 lung (367 adenocarcinomas (AC), 13 adenosquamous carcinomas, 82 bronchioloalveolar carcinomas, 258 large cell carcinomas, 63 neuroendocrine carcinomas, 744 squamous cell carcinomas (SCC)) and 553 prostate carcinomas were then arrayed as described before (Bubendorf *et al*, 2001). Briefly, tissue cylinders with a diameter of 0.6 mm were punched from representative tumour areas of each donor tissue block and brought into a recipient paraffin block. Multiple 4 *μ*m sections of the resulting tissue microarray block were cut and mounted to an adhesive-coated slide system.

### Clinical data

All relevant patient data were anonymised. Sex, age and stage according to the WHO/UICC 1997 were recorded in subsets of gastric, colon, lung and prostate cancers. In prostate cancers, the Gleason score was available. Grade was also known in colon and lung cancers. Additionally, the survival time was recorded in tumours from the colon, lung and prostate, but not stomach. Overall survival (OS) was calculated from the date of diagnosis until death from any cause or date of last contact for living patients. The cause of death was recorded in a subset of patients to calculate the tumour-specific survival.

### Immunohistochemistry

Standard indirect staining procedures were used for immunohistochemistry (ABC-Elite-Kit, Vector Laboratories, Burlingame, CA, USA). After heat-induced pretreatment (water bath, 30 min, 99°C in target retrieval solution buffer (DAKO code S1699, DAKO, Glostrup, Denmark)) for antigen retrieval, a mouse monoclonal anti-Ep-CAM-antibody (ESA, clone VU-1D9, Novocastra, Newcastle upon Tyne, UK) was applied for 2 h at a dilution of 1 : 50 at 37°C. The slides were then incubated with the secondary, biotinylated antibody. Osmium-enhanced diaminobenzidine was used as the chromogen. Counterstaining was carried out with Harris' haematoxylin. Only fresh cut slides were stained simultaneously to minimise the influence of slide ageing and maximise repeatability and reproducibility of the experiment. For negative controls, the primary antibody was omitted, as positive controls the internal normal tissues with known EpCam positivity were used. For each sample, staining intensity (0, faint to moderate, intense) and percentage of positive tumour cells was estimated. A case was considered strongly positive if the antibody detected >70% positive cells, otherwise weakly positive, or negative if no cells were stained. Staining intensity was recorded, but not used for correlation with clinical findings, as it can vary depending on the manner of tissue fixation. Cytoplasmic staining alone was considered as nonspecific as Ep-CAM is localised on the cell membrane. These cases were excluded from analysis. The slides were all evaluated in one day by one experienced pathologist (GS) to minimise inter- and intraobserver variability of the results.

### Statistics

The software used for statistical analysis was statview 5.0 (SAS Institute Inc., NC, USA) The Fisher's exact test and the *χ*^2^ test were used to compare Ep-CAM expression and clinical and morphological tumour characteristics. For survival analysis, patients with Ep-CAM weakly to moderately positive and negative tumours were grouped together to emphasise on Ep-CAM overexpressing tumours. Survival curves were plotted according to Kaplan–Meier. The univariate association between individual clinical features and overall survival (OS) was determined with the log-rank test. Factors independently associated with OS were identified in a multivariate analysis by the Cox proportional hazards regression model. The limit of significance for all analyses was defined as a *P*-value <0.05; two-sided tests were used in all calculations.

## RESULTS

**Figure 1:**
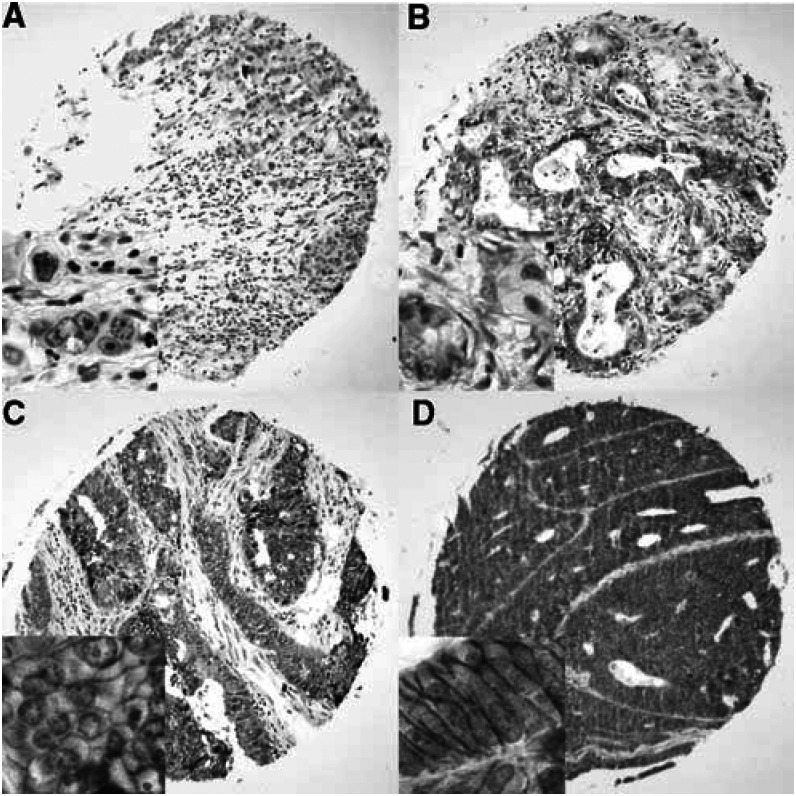
Examples of Ep-CAM staining of four human colon carcinoma samples from a tissue microarray. (**A**) Sample with no Ep-CAM staining. Samples with weak (**B**), moderate (**C**) or intense (**D**) Ep-CAM staining. Note that in (**B**) and (**C**) the staining intensity and quantity of positive tumour cells are different. Inlets showing detailed view of the membrane localised staining product.

Clinical information was available for 3746 tumours (Table 1), whereas Ep-CAM staining results from tissue microarrays were obtained for 3360 tumour samples. A total of 686 tumour samples (17%) on microarrays could not be analysed due to issues of sample quality. Figure 1 shows examples for different intensities of Ep-CAM-specific immunohistochemical staining of tissue microarray samples from four colon cancer patients.

In all four tumour entities, staining was predominantly membranous but cytoplasmic staining could also be seen in cases with an intense staining. In total, 74.1% of the 3360 tumours showed a high-level Ep-CAM staining. In total, 85.1% of samples showed a staining in >70% of tumour cells. Of note, 92.2% (2118 of 2297 cases) of tumours with an Ep-CAM-positive cell fraction >70% had at the same time the highest staining intensity score, indicating a marked coincidence of high-level staining intensity with a high fraction of positive tumour cells. As intensity is typically subdued to variations following tissue fixation and most notably staining procedures, the equal high staining intensity in the majority of cases well reflects the highly optimised staining procedure, whereas the high percentage of positive cases indicates an excellent preservation of the investigated antigen in the samples selected for construction of microarrays. This obviously led to an almost binary data set in which only 11.6% of the 3360 tumour samples showed a positive tumour cell fraction of less than 70%, and on average 19.6% a weak to moderate staining intensity. The observed ‘black or white’ pattern was further emphasised by the scoring system, correlating only strongly positive tumours with survival data. On average, Ep-CAM expression was completely absent from only 5.9% of tumours (198 cases) based on immunohistochemical analysis. An overview of staining results across all tumour samples is shown in Table 2.

### Epithelial cell adhesion molecule expression in colon cancer

In the colon cancer microarray (Table 3), samples from 1186 patients were analysable. Most of the cases showed an intense staining signal in the vast majority of tumour cells. Only seven tumours (0.6%) showed a faint staining intensity, whereas 1152 tumours (97.1%) showed a strong staining intensity. In total, 97.7% of cases (*n*=1159) showed Ep-CAM expression in more than 70% of tumour cells.

Highly differentiated colon cancers expressed Ep-CAM significantly more frequently and strongly than the other colon cancers (*P*<0.0001). However, the low differentiated colon cancers of grade 3 were still strongly positive for Ep-CAM in 92.1% of cases.

In an univariate survival analysis, the lymph node status (pN0 *vs* pN+, *P*<0.0001), vascular invasion (*P*<0.0001) and postoperative chemotherapy (*P*<0.0001) were significant regarding tumour-specific survival. Because Ep-CAM expression and tumour grade showed a significant association, the different grades were analysed separately. Patients with Ep-CAM negative, moderately differentiated colon cancers (Grade 2) showed a significantly inferior tumour-specific survival (OR 5.421, 95% CI 1.685–17.442, *P*=0.0014, *n*=284), whereas in the other subgroups patients with strongly Ep-CAM expressing tumours showed no such trend towards a better survival. This association in the G2 colon carcinomas remained significant in a multivariate analysis including Ep-CAM expression (OR 11.175, 95% CI 3.327–37.534, *P*<0.0001), the lymph node status (OR 3.169, 95% CI 1.768–5.680, *P*=0.0001), vascular invasion (OR 2.408, 95% CI 1.345–4.309, *P*=0.0031), whereas postoperative chemotherapy (OR 0.772, 95% CI 0.421–1.413, *P*=0.4006) showed no statistical significance.

## EPITHELIAL CELL ADHESION MOLECULE EXPRESSION IN STOMACH CANCER

On the stomach cancer microarray (Table 4), 473 cases were analyzable. In total, 90.7% of the tumours (*n*=429) expressed Ep-CAM on >70% of cells and 85.8% of the cases (*n*=406) showed the highest level of staining intensity. Epithelial cell adhesion molecule frequency was lowest in pT4 tumours (77.8%), while all other subgroups showed Ep-CAM expression in more than 80% of tumours. No significant correlation of Ep-CAM expression between primary tumour, nodal or metastasised stage was found. In this group of patients, no data on patient survival were available for correlation with Ep-CAM expression.

### Epithelial cell adhesion molecule expression in prostate cancer

In the prostate cancer microarray, 414 cases were analyzable (Table 5). Of these, 361 cases (87.2%) showed a strong Ep-CAM expression. Epithelial cell adhesion molecule expression and stage or grade according to Gleason did not correlate. In univariate survival analysis, there was the expected significant correlation between Gleason score and survival in (*P*<0.0001). However, there was no correlation of Gleason score or survival with Ep-CAM expression.

### Epithelial cell adhesion molecule expression in lung cancers

In the lung cancer array, 1287 cases were analyzable (Table 6). On average, 64% of cases (*n*=823) showed a high Ep-CAM expression score. An intense staining signal was detected in 51% of cases (*n*=660). Squamous cell (53.6%, *n*=332), neuroendocrine (64.3%, *n*=36) and large cell carcinomas (67%, *n*=150) were expressing Ep-CAM less frequently at a high level than adenocarcinomas (80.8%, *n*=256) (*P*<0.0001). There was no association of stage or grade and Ep-CAM expression in any of the histological subgroups.

In univariate survival analysis, the expected significant correlation between tumour stage and survival was observed (*P*<0.0001). Because the different histological subgroups showed varying Ep-CAM expression, they were analysed separately. No definite correlations were found regarding Ep-CAM expression, grade and survival in any of the histological entities, although sample bias cannot be completely excluded as our tumour collective did not contain poorly differentiated tumours. There was a trend toward a longer survival in patients with adenocarcinomas, large cell and bronchioloalveolar carcinomas and strong Ep-CAM expression. This trend was inversed in SCC.

## DISCUSSION

This is the largest analysis of Ep-CAM expression in major human malignancies performed to date. It employed a highly reproducible and standardised high-throughput array technology, which allows comparison of staining intensity and frequency for a large set of tissue samples. Comparability of IHC data within and across studies is frequently hampered by the use of distinct fixation, staining and antibody detection protocols. In addition, different antibodies are used in the literature to stain a particular target antigen. In the present analysis, all these parameters were kept constant. The use of high sample numbers, one of the main advantages of array technology, resulted in reaching statistically meaningful conclusions. With lung, colon, prostate and gastric cancers, we have selected four of the most frequent cancers in the industrialised world. Epithelial cell adhesion molecule expression in breast cancer has been previously published for more than 1700 patient samples (Gastl *et al*, 2000; Spizzo *et al*, 2002; Spizzo *et al*, 2004).

In primary tumours of colon, lung, prostate and stomach, Ep-CAM was on average significantly expressed in 94.1% of 3360 cases. In our analysis, Ep-CAM expression showed a tendency to be binary with the larger group consisting of tumours with high staining intensity on a major fraction of tumour cells, and a much smaller group expressing Ep-CAM only at weak or moderate levels, or not all. In well and poorly differentiated colon cancers, and lung and prostate cancer, no obvious correlation of Ep-CAM expression with survival, tumour stage or grade was observed. Gastric cancer could not be analysed for a correlation of Ep-CAM expression with survival because no patient survival data were available for this indication. Previous studies investigated the influence of Ep-CAM expression on survival in a number of other carcinoma. Epithelial cell adhesion molecule upregulation was an independent marker for poor survival in lymph node positive breast cancers (Spizzo *et al*, 2004), and gall bladder cancers (Varga *et al*, 2004). In contrast, improved survival was found associated with Ep-CAM upregulation in clear cell renal cell carcinoma (Kim *et al*, 2004; Seligson *et al*, 2004; Went *et al*, 2005), and gastric cancers (Songun *et al*, 2005). Here, we observed that survival of patients in the subgroup of moderately differentiated colon cancers also showed a significant positive correlation with Ep-CAM expression. Consistent with previous reports, we could not find an influence of Ep-CAM expression on patients' survival outcome in lung and prostate cancers (Poczatek *et al*, 1999; Piyathilake *et al*, 2000).

How can the effect of Ep-CAM overexpression on survival be so different depending on cancer indication, and for subgroups within a given indication? The emerging functional importance of Ep-CAM for tumour cells would be more consistent with the phenotype of breast and gall bladder cancers, where Ep-CAM overexpression is an independent predictor of poor survival (Spizzo *et al*, 2004; Varga *et al*, 2004). Overexpressed Ep-CAM was shown to provide a potent growth stimulus to tumour cells enabling proliferation (Munz *et al*, 2004), and led to an increased invasiveness and migration of tumour cells presumably due to E-cadherin antagonism (Litvinov *et al*, 1997; Osta *et al*, 2004). It is likely that Ep-CAM exerts the same functions in cells of primary tumours and metastases, which would well explain why Ep-CAM shows high-level and largely uniform expression on tumour cells of most patients with adenocarcinoma of lung, prostate, breast, colon, and gastric cancer. In many of these cancers, the fraction of Ep-CAM-negative tumours is very small. For instance, in the present study, only four out of 1186 colon cancer samples were Ep-CAM-negative. In breast cancer, the analytical situation was better because approximately 10% of tumours are Ep-CAM negative (Spizzo *et al*, 2004). Hence, despite very large sample numbers, it may be still difficult to accrue large enough sample populations for statistical comparison of Ep-CAM-negative with positive tumours. As a consequence, studies have to rely on comparing different expression levels of Ep-CAM, the accuracy of which is limited by the semiquantitative nature of immunohistochemical staining procedures.

A hallmark of tumour cells is de-differentiation, which typically goes along with the loss of expression of differentiation markers (Kiemer *et al*, 2001; Peinado *et al*, 2004). Epithelial cell adhesion molecule is an epithelial differentiation marker, which is frequently expressed on normal epithelial cells (Winter *et al*, 2003). Loss of Ep-CAM expression is therefore a likely consequence of tumour cell de-differentiation, as for instance is seen for the epithelial differentiation antigen E-cadherin (Sulzer *et al*, 1998). On the other hand, overexpression or maintenance of Ep-CAM expression on tumour cells relative to normal epithelia may then be an indication that its presence confers a benefit to tumour cells. The level of Ep-CAM expression on tumour cells will then reflect a balance of reduced expression due to de-differentiation and over- or maintained expression as a consequence of positive selection of a certain growth phenotype. The survival difference between patient populations with high and low Ep-CAM-expressing tumours should therefore be seen in light of an antagonism between de-differentiation and positive selection.

While both high and low Ep-CAM-expressing tumours may still benefit from growth-stimulatory and metastatic properties of Ep-CAM, the population of low and no Ep-CAM expressors may have undergone further de-differentiation events to a level were reduced expression or loss of Ep-CAM has been compensated for by overexpression of other growth-promoting proteins. Examples are growth factor receptors like HER-2 and EGFR, which in contrast to Ep-CAM, are upregulated as a consequence of gene amplification events (Simon *et al*, 2003; Al-Kuraya *et al*, 2004). Future studies are needed to explore whether reduced Ep-CAM expression or its loss is linked to a concomitant overexpression of other proteins with oncogenic potential. If this functional compensation is specific for tumour type, it could explain why Ep-CAM expression has such distinct effects on survival prognosis over different indications.

Its high-level, frequent and homogenous expression on human adenocarcinoma make Ep-CAM an ideal target for antibody-based immunotherapeutic approaches. Epithelial cell adhesion molecule is currently targeted by two principally different approaches in cancer therapy: passive and active immunotherapy. The first antibody used in passive immunotherapy was edrecolomab, a murine IgG2a antibody targeting Ep-CAM (Sears *et al*, 1984; Riethmuller *et al*, 1994). The therapeutic effect of this antibody administered alone or in combination with chemotherapy or GM-CSF (Mellstedt *et al*, 2000) was not conclusive, but it showed a very benign safety profile (Fields *et al*, 2002; Punt *et al*, 2002; Hartung *et al*, 2005). To reduce immunogenicity and enhance antibody-dependant cytotoxicity, complement dependant cytotoxicity and serum half-life, humanised antibodies ING-1 (de Bono *et al*, 2004), 3622W94 (Abdullah *et al*, 1999; Martin *et al*, 1999) and fully human IgG1 antibody MT201 (adecatumumab) (Naundorf *et al*, 2002; Brischwein *et al*, 2005) were developed. All showed much higher *in vitro* cytotoxic activity than edrecolomab, but the two high-affinity antibodies ING-1 and 3622W94 turned out to be much less tolerable than edrecolomab due to induction of acute pancreatitis. Conjugation of the Ep-CAM-specific murine monoclonal antibody 323/A3 human with beta-glucuronidase is a prodrug approach designed to locally augment the anti-tumour effect of doxorubicin (Houba *et al*, 2001). A fusion protein between a single-chain antibody and a bacterial toxin is currently being tested for local treatment of head and neck tumours in a phase I trial (Quenneville *et al*, 2005), and has shown extraordinary antitumour activity and potency in a xenograft model (Di Paolo *et al*, 2003). Epithelial cell adhesion molecule was also selected as target for bi- and trispecific antibody therapies. Three T-cell activating, single-chain bispecific antibodies were shown to potently eradicate established and disseminated tumours in immunodeficient and -competent mouse models (Peters *et al*, 2004; Brischwein *et al*, 2005; Schlereth *et al*, 2005a, 2005b), and a related bispecific antibody called E3Bi demonstrated high *in-vitro* cytotoxicity (Ren-Heidenreich *et al*, 2004). The trifunctional antibody catumaxomab (anti-Ep-CAM × anti-CD3) has been safely administered in a phase I/II study to patients suffering from malignant ascites (Stroehlein *et al*, 2005). Bispecific antibodies that link adenovirus to Ep-CAM are experimentally used in combination with recombinant adenoviral vectors for cancer gene therapy (Haisma *et al*, 1999), and adenoviral vectors expressing virus with an anti-Ep-CAM surface protein were also constructed (Oosterhoff *et al*, 2005).

One active immunotherapy approach is using IGN101 (formulated edrecolomab) for induction of anti-Ep-CAM anti-idiotypic antibodies (Zaloudik *et al*, 2002). This vaccination was shown to be safe, reduce Ep-CAM positive tumour cells in circulation and prolong survival of patients with metastatic rectal cancer (Himmler *et al*, 2005). Current experimental approaches are using the Ep-CAM promoter to control the expression of therapeutic genes (Gires *et al*, 2004), and short interfering RNA for the silencing of Ep-CAM expression, which resulted in a 35–80% decrease in proliferation of breast cancer cell lines (Osta *et al*, 2004). Lastly, the Ep-CAM protein is being used as a vaccine to elicit by various approaches the induction of specific T and B cell responses (Mosolits *et al*, 2004, 2005; Neidhart *et al*, 2004).

The emerging results from Ep-CAM-targeting immunotherapies show a wide utility of the target antigen. Although Ep-CAM is also expressed on normal epithelia, a lower expression there compared to tumour cells (Kim *et al*, 2004; Osta *et al*, 2004) and a possible sequestration of Ep-CAM between epithelial cells (McLaughlin *et al*, 2001) appears to open a therapeutic window. This is supported by the benign safety profile of certain Ep-CAM targeted therapies such as edrecolomab, adecatumumab and vaccination approaches with the autoantigen. In indications showing a strong correlation between Ep-CAM overexpression and poor survival prognosis, selective ablation of Ep-CAM positive cells by the above therapeutic approaches may translate into a survival benefit. In indications with a neutral or positive effect of Ep-CAM overexpression on survival, the high proportion of Ep-CAM positive cells in tumours may still be efficacious in particular if the targeted immunotherapy is combined with chemotherapeutics, and if used in adjuvant settings as are characterised by low tumour load.

## Figures and Tables

**Table 1:** Clinical characteristics of analysed patients and tumors

	**Carcinoma**				
	**Lung**	**Colon**	**Stomach**	**Prostate**	**All**
Number	1534	1186	473	553	3746
					
*Sex*					
Female	201	735	117	0	1053
Male	826	670	311	553	2360
					
Median age	61.9	69.7	59	64.7	
(years, range)	(29–87)	(36–96)	(27–92)	(45–92)	
Survival	36.4	50.5		82.5	
(months, range)	(0–200)	(0–152)		(0–345)	
					
*Stage*					
pT1	276	62	3	36	377
pT2	864	202	39	401	1506
pT3	250	892	362	88	1592
pT4	104	222	10	22	358
					
*Nodal stage*					
pN0	776	703	153	509	2141
pN1	335	355	245	14	949
pN2	338	292	64	1	695
pN3	39	0	7	0	46
					
*Metastases*					
pM0	1394	NA	445	542	2381
pM1	94	NA	25	0	119
					
*Grade (Gleason score in prostate carcinoma)*					
1	140	31	NA		
2	362	1176	NA		
3	0	177	NA	3	
4				17	
5				134	
6				169	
7				191	
8				17	
9				19	
10				2	

Stage, nodal stage and metastases according to the WHO/UICC (1997).

NA: Not available.

**Table 2:** Frequency of Ep-CAM overexpression in major human cancers

		**Ep-CAM expression**		
**Tumour entity**	***n***	**Negative (%)**	**Weak/ moderate** **(%)**	**Strong (%)**
All	3360	5.9	11.6	82.5
				
*Colon*				
Adenocarcinoma NOS	1086	0.3	1.7	98.0
Medullary carcinoma	5	20.0	0	80.0
Mucinous carcinoma	88	0	4.5	95.5
Signet ring cell carcinoma	4	0	0	100.0
Other types	3	0	0	100.0
				
*Stomach*				
Stomach carcinoma	473	2.5	6.8	90.7
				
*Lung*				
Adenocarcinoma NOS	317	5.3	13.9	80.8
Adenosquamous carcinoma	11	45.4	18.2	36.4
Bronchioloalveolar carcinoma	60	3.3	21.7	75.0
Large cell carcinoma	224	15.1	17.9	67.0
Neuroendocrine carcinoma	56	16.1	19.6	64.3
Squamous cell carcinoma	619	17.3	29.1	53.6
				
*Prostate*				
Prostate carcinoma	414	1.9	10.9	87.2

**Table 3:**
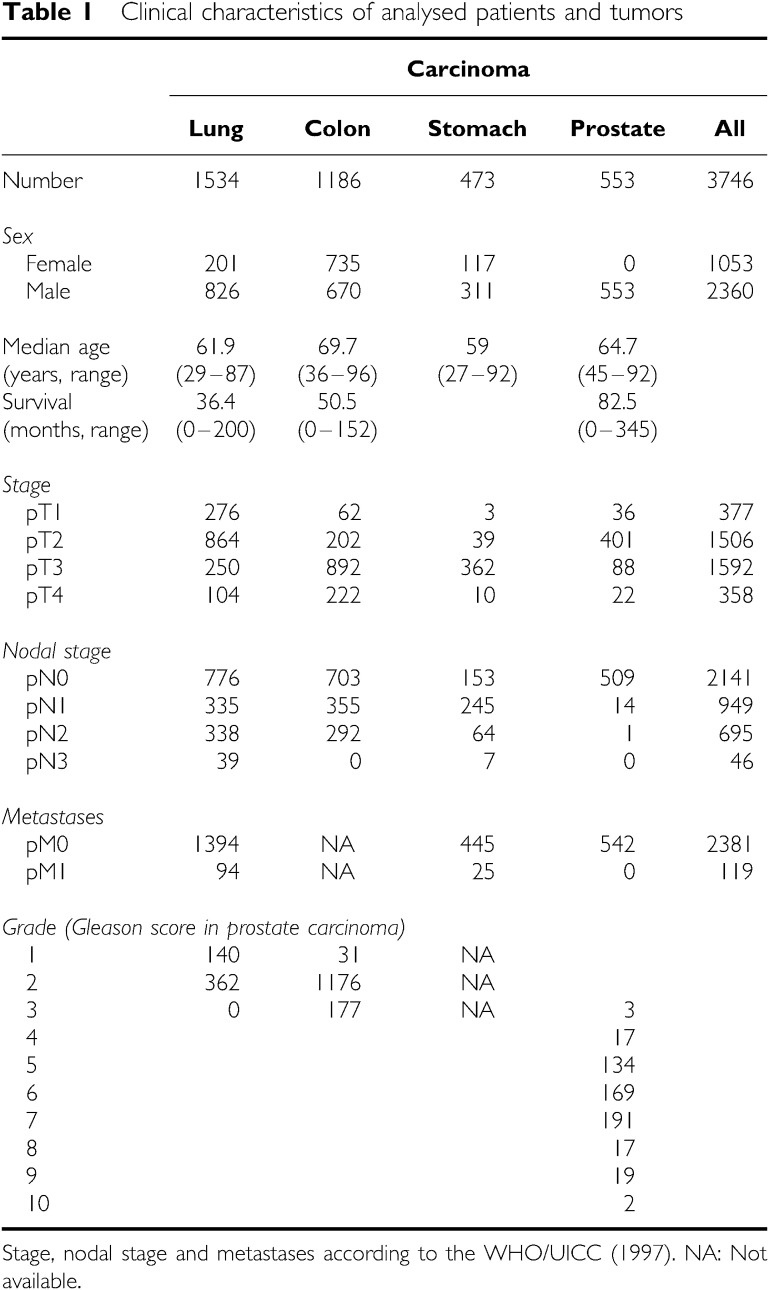
Expression of Ep-CAM in colon carcinoma

**Table 4:**
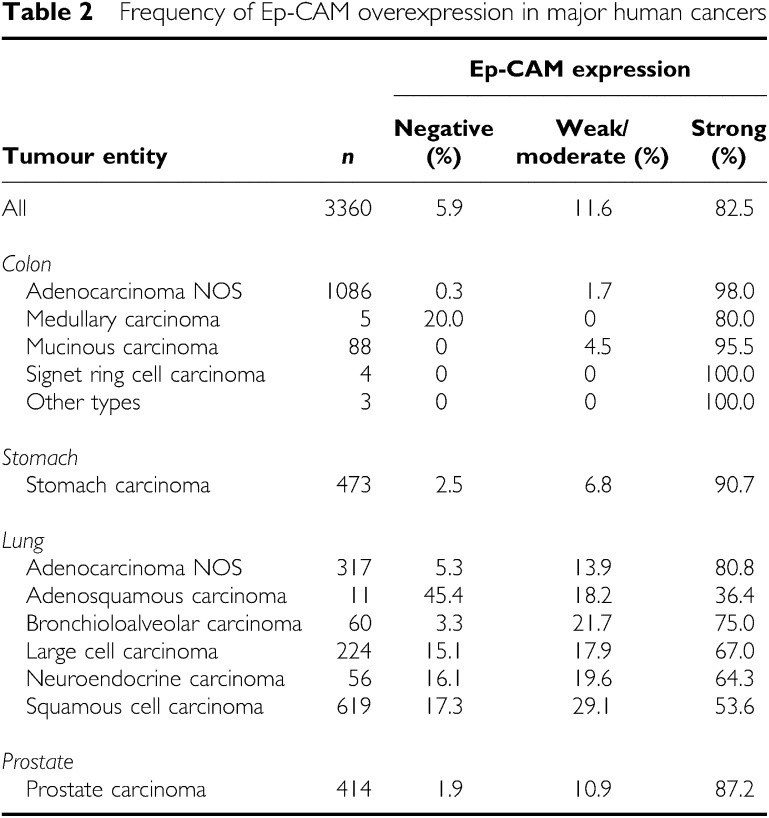
Expression of Ep-CAM in gastric carcinoma

**Table 5:**
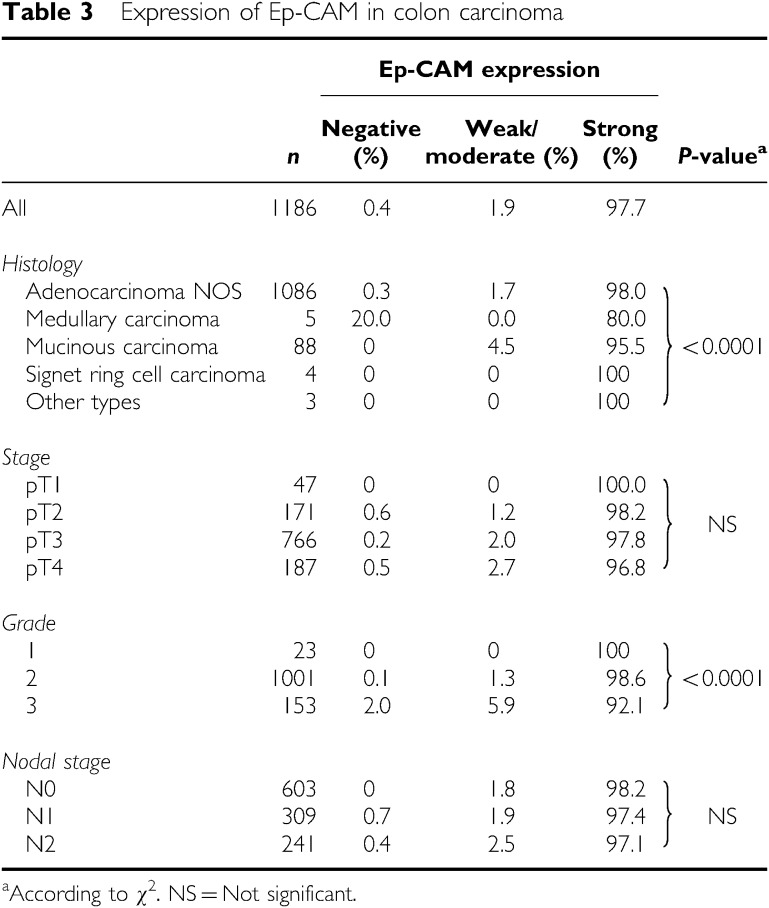
Expression of Ep-CAM in prostate carcinoma

**Table 6:**
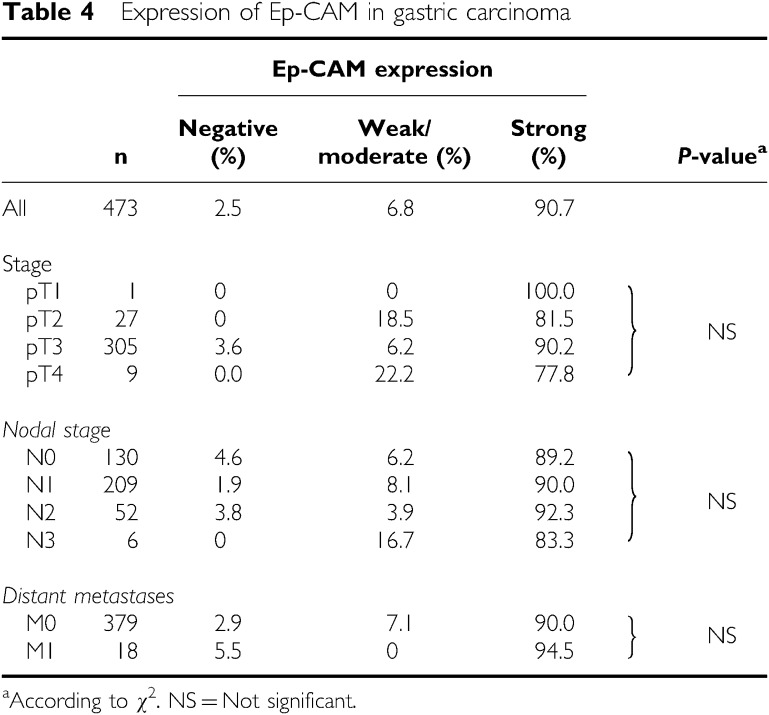
Expression of Ep-CAM in lung carcinoma
